# Gene silencing in the aedine cell lines C6/36 and U4.4 using long double-stranded RNA

**DOI:** 10.1186/s13071-024-06340-3

**Published:** 2024-06-11

**Authors:** Bodunrin Omokungbe, Alejandra Centurión, Sabrina Stiehler, Antonia Morr, Andreas Vilcinskas, Antje Steinbrink, Kornelia Hardes

**Affiliations:** 1 LOEWE Centre for Translational Biodiversity Genomics (LOEWE TBG), Senckenberganlage 25, 60325 Frankfurt Am Main, Germany; 2 Institute for Insect Biotechnology, Justus-Liebig University, Heinrich-Buff-Ring 26-32, 35392 Giessen, Germany; 3 Fraunhofer Institute for Molecular Biology and Applied Ecology IME, Branch of Bioresources, Ohlebergsweg 12, 35392 Giessen, Germany; 4 BMBF Junior Research Group in Infection Research “ASCRIBE”, Ohlebergsweg 12, 35392 Giessen, Germany

**Keywords:** RNAi, dsRNA, Transfection reagents, *Aedes albopictus*, Vector, Semliki Forest virus, Arbovirus, mCherry, Cell culture

## Abstract

**Background:**

RNA interference (RNAi) is a target-specific gene silencing method that can be used to determine gene functions and investigate host–pathogen interactions, as well as facilitating the development of ecofriendly pesticides. Commercially available transfection reagents (TRs) can improve the efficacy of RNAi. However, we currently lack a product and protocol for the transfection of insect cell lines with long double-stranded RNA (dsRNA).

**Methods:**

We used agarose gel electrophoresis to determine the capacity of eight TRs to form complexes with long dsRNA. A CellTiter-Glo assay was then used to assess the cytotoxicity of the resulting lipoplexes. We also measured the cellular uptake of dsRNA by fluorescence microscopy using the fluorophore Cy3 as a label. Finally, we analyzed the TRs based on their transfection efficacy and compared the RNAi responses of *Aedes albopictus* C6/36 and U4.4 cells by knocking down an mCherry reporter Semliki Forest virus in both cell lines.

**Results:**

The TRs from Biontex (K4, Metafectene Pro, and Metafectene SI+) showed the best complexing capacity and the lowest dsRNA:TR ratio needed for complete complex formation. Only HiPerFect was unable to complex the dsRNA completely, even at a ratio of 1:9. Most of the complexes containing mCherry-dsRNA were nontoxic at 2 ng/µL, but Lipofectamine 2000 was toxic at 1 ng/µL in U4.4 cells and at 2 ng/µL in C6/36 cells. The transfection of U4.4 cells with mCherry-dsRNA/TR complexes achieved significant knockdown of the virus reporter. Comparison of the RNAi response in C6/36 and U4.4 cells suggested that C6/36 cells lack the antiviral RNAi response because there was no significant knockdown of the virus reporter in any of the treatments.

**Conclusions:**

C6/36 cells have an impaired RNAi response as previously reported. This investigation provides valuable information for future RNAi experiments by showing how to mitigate the adverse effects attributed to TRs. This will facilitate the judicious selection of TRs and transfection conditions conducive to RNAi research in mosquitoes.

**Graphical Abstract:**

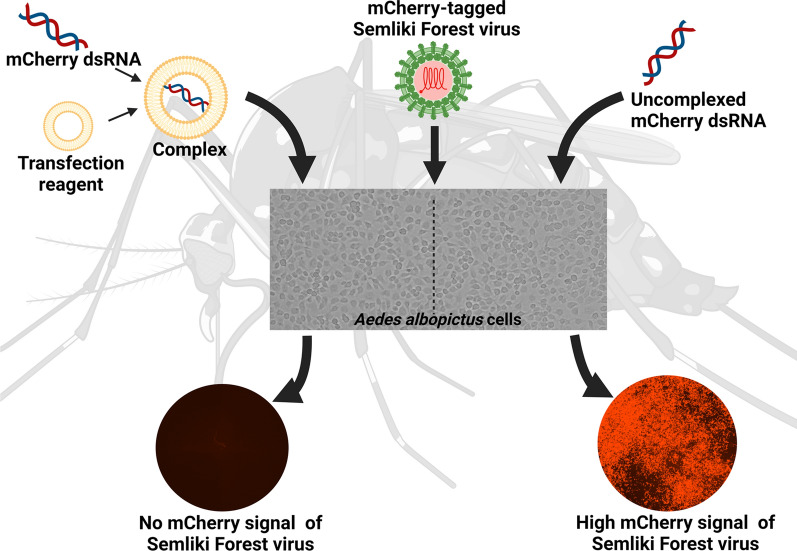

**Supplementary Information:**

The online version contains supplementary material available at 10.1186/s13071-024-06340-3.

## Background

Mosquitoes transmit many pathogens, including Zika virus (ZIKV), West Nile virus (WNV), dengue virus (DENV), chikungunya virus (CHIKV), *Mycobacterium ulcerans*, and malaria parasites [1, 2]. There are no vaccines or specific treatments available for the diseases caused by most of these pathogens [3–5]. Vector control is therefore needed to reduce the transmission risk of vector-borne diseases [6]. However, the efficacy of conventional vector-control methods is decreasing due to the emergence of resistant mosquito populations [7]. Furthermore, some chemical insecticides have been banned due to their adverse effects on nontarget insects, livestock animals, and humans [8]. In this regard, RNA interference (RNAi) is a powerful research tool for the analysis of gene functions and host–pathogen interactions that can also be exploited for the development of ecofriendly pesticides [9, 10].

RNAi is an evolutionarily conserved process in eukaryotes in which RNA molecules trigger posttranscriptional gene silencing [11, 12]. Efficient RNAi pathways are present in plants [13], nematodes [14], fungi [15], and insects [16]. In mosquitoes, the three RNAi pathways identified thus far are those based on microRNA (miRNA), Piwi-interacting RNA (piRNA), and small interfering RNA (siRNA) [17]. The siRNA machinery mainly provides defense against exogenous nucleic acids and transposable elements by targeted gene suppression [18, 19]. This pathway is triggered by the detection of cytoplasmic double-stranded RNA (dsRNA), which is cleaved into ~21-nucleotide (nt) siRNAs by Dicer-2 and R2D2 and then loaded onto an siRNA-induced silencing complex (siRISC) containing the protein Argonaute 2 (Ago2). Here, the siRNA is separated into single strands, one of which is retained to guide the siRISC to complementary target RNA sequences that are ultimately cleaved and degraded [16, 17, 20]. However, open questions in regards to the replication properties of some arboviruses, their interaction with innate immunity, and RNAi machinery remain [17].

The potential of RNAi as a control strategy against insect pests was first shown in beetles [21] and moths [22]. Prominent targets include nucampholin (NCM), Ras opposite (ROP), RNA polymerase II subunit-140 (RPII-140), and dre4, which were effective for the control of the red flour beetle *Tribolium castaneum* [23, 24]. In *Aedes albopictus*, potential target genes for RNAi-based control in mosquitoes include chitin synthase, β-tubulin, and the inhibitor of apoptosis (see [25] for a review). RNAi has been used to study 18 *Aedes aegypti* carboxypeptidase genes, where quantitative expression revealed that 11 of the genes were induced up to 40-fold in the midgut in response to blood feeding [26]. RNAi has also been used to study DENV and insect-specific flavivirus cell fusing agent virus (CFAV) in *Ae. albopictus* C6/36 cells and *Ae. aegypti* Aag2 cells. The production of siRNA was detected in Aag2 cells, whereas C6/36 cells demonstrated suboptimal Dcr2 cleavage efficiency when exposed to long dsRNA [27]. Target-specific dsRNA was also used to understand virus–host interactions and to inhibit the replication of Semliki Forest virus (SFV) in *Ae. albopictus* U4.4 cells [28].

The efficacy of RNAi is affected by factors such as dsRNA stability and internalization, the functionality of the RNAi machinery, systemic spreading of the RNAi signal, and the suitability of target genes [29]. The stability of dsRNA can be influenced by the presence of dsRNases that cause rapid degradation and also by the pH, because the optimal pH of dsRNA is 4.0–5.0 [29, 30]. The stability of dsRNA may therefore differ in the mosquito midgut, saliva, and hemolymph because the pH ranges from 7.5 to 11.0 [31, 32]. To overcome some of these barriers, dsRNA can be introduced using carrier systems such as cell-penetrating peptides [11], polymers [33], liposome-based transfection reagents (TRs) [34], and other nanoparticles [35]. These carriers can facilitate the transport of dsRNA into cells while protecting the cargo from degradation by dsRNases and pH changes [30].

TRs are designed to facilitate the introduction of nucleic acids into eukaryotic cells and usually feature a positively charged head group and one or two hydrocarbon chains that bind to negatively charged nucleic acids via electrostatic interactions to form cationic complexes (also known as lipoplexes). These complexes interact with the negatively charged phospholipid bilayer of the cell membrane, promoting uptake and intracellular release [36–39]. The formulation and composition of most commercially available TRs is not disclosed [40], making it difficult to rationally select TRs based on their components. Furthermore, certain TR components may be cytotoxic, thus affecting the reliability of transfection results [41, 42]. For example, six commercially available TRs (Arrest-In, ExpressFect, FuGENE HD, jetPEI, Lipofectamine 2000, and SuperFect) were examined based on their transfection efficiency and cytotoxicity using nine mammalian cell lines, revealing that FuGENE HD was most efficient for many of the cell lines, followed by Arrest-In and jetPEI, but jetPEI and ExpressFect were the least cytotoxic [41]. Currently, there are no commercially available TRs specifically designed for the introduction of long dsRNA (defined as dsRNA exceeding 300 bp [43]) and to our knowledge a comparative study assessing the efficiency of various TRs in aedine cells has not been published.

Cell culture can be used as a preliminary screening tool in RNAi studies to assess the feasibility, efficacy, and specificity of RNAi constructs before transferring to in vivo experiments, optimizing the use of resources and minimizing ethical concerns [44]. We focused on the use of TRs (Table 1) from different manufacturers as dsRNA carrier systems for the introduction of long dsRNA into the aedine cell lines C6/36 and U4.4. We assessed the TRs according to their complexing capacity, cytotoxicity, impact on the uptake of dsRNA, and overall efficacy of the TRs. We also evaluated the suspected lack of an antiviral RNAi machinery in C6/36 cells [45]. Our results indicate the best conditions for testing dsRNA in aedine cells using various commercially available TRs and will facilitate RNAi research, e.g., the development of ecofriendly pesticides.

**Table 1:** List of commercially available transfection reagents used for the analysis of complexing capacity, including their concentration and parameters used for complex formation

Transfection reagent	Manufacturer	Concentration (mg/mL)	Dilution medium	Incubation time (min)
K4 Transfection System	Biontex	1.5	Grace’s insect medium	20
Metafectene Pro	Biontex	1.5	Grace’s insect medium	20
Metafectene SI+	Biontex	1.5	Grace’s insect medium	20
Lipofectamine 2000	Invitrogen	1.0	OptiMEM reduced serum medium	5 + 20
Lipofectamine RNAiMAX	Invitrogen	–	OptiMEM reduced serum medium	5
CellFectin II	Invitrogen	1.0	Grace’s insect medium	20
SiLentFect	BioRad	1.0	Grace’s insect medium	20
HiPerFect	Qiagen	–	Grace’s insect medium	10

## Methods

### Cell culture

The *Ae. albopictus* cell lines C6/36 (kindly provided by Prof. Dr. Stefanie Becker) and U4.4 (Friedrich-Loeffler-Institute, Federal Research Institute for Animal Health, Greifswald, Riems, Germany) were cultured in insect cell growth medium (L15 medium GlutaMax) supplemented with 1% tryptose phosphate broth, 10% fetal calf serum, 1% MEM nonessential amino acids, and 1% penicillin/streptomycin at 28 °C. Baby hamster kidney (BHK-21) cells (CLS Cell Lines Service, Eppelheim, Germany) were maintained in mammalian cell growth medium (DMEM GlutaMax) supplemented with 10% fetal calf serum and 1% penicillin/streptomycin at 37 °C in a 5% CO_2_ atmosphere. All media and supplements were from Thermo Fisher Scientific (Frankfurt, Germany).

### Preparation of dsRNA

A glycerol stock of *Escherichia coli* NEB 5-α (New England BioLabs, Frankfurt, Germany) carrying vector pCMV-SFV6-2SG-mCherry was inoculated into 5 mL sterile lysogeny broth (LB) containing 125 µg kanamycin and incubated overnight at 37 °C, shaking at 200 rpm. Plasmid DNA was isolated using the NucleoSpin Plasmid DNA kit (Macherey–Nagel, Düren, Germany) according to the manufacturer’s protocol. Gene-specific primers linked to a T7 promoter were used to amplify a part of the mCherry region from the SFV6-2SG-mCherry genome with OneTaq Hot Start Quick-Load 2× Master Mix (New England BioLabs) according to the manufacturer’s protocol. The PCR products were used to synthesize the dsRNA in vitro using the MEGAscript T7 Transcription kit (Thermo Fisher Scientific). The dsRNA was purified by LiCl precipitation and resuspended in nuclease-free water. The concentration of the dsRNA was determined using a Nanodrop 2000 spectrophotometer (Thermo Fisher Scientific) with factor 46.0 and was stored at −80 °C. For dsRNA targeting green fluorescent protein (GFP), we followed the same procedure but used a glycerol stock of *E. coli* carrying vector pGEM-T-Easy-GFP-125 and gene-specific primers targeting GFP linked to the T7 promoter. See Table S1 in Additional file 1 for primer sequences and dsRNA sequences.

### Complexation of dsRNA using commercially available transfection reagents

To determine the complexing capacity of TRs and the ratio needed to form complete complexes with mCherry-dsRNA, the TRs and dsRNA were diluted to 0.2 mg/mL (Table 1). The concentration of Lipofectamine RNAiMAX and HiPerFect were not provided by the manufacturers, so we equated these TRs to be 1.0 mg/mL. The components were mixed at dsRNA:TR ratios ranging from 1:0.3 to 1:9 (ratios adjusted according to the complexing capacity) and incubated at room temperature for the appropriate time (Table 1). Immediately after incubation, Mass Ruler loading dye (Thermo Fisher Scientific) was added to each complex and the complexes were resolved by 1.5% (w/v) agarose gel electrophoresis using pulse-field certified agarose (Bio-Rad Laboratories, Munich, Germany) for 80 min at 110 V and 150 mA in a Bio-Rad Sub-Cell GT (Bio-Rad Laboratories). The gel was visualized on Gel Doc XR+ using ImageLab v5.2.1 (both Bio-Rad Laboratories).

### Cytotoxicity of the dsRNA/TR complexes in C6/36 and U4.4 cells

Cells were seeded in 96-well plates and treated at ~50% confluency with mCherry-dsRNA complexed with different TRs at concentrations ranging from 0.5 to 2 ng/µL, using the optimal dsRNA:TR ratios (Table 2). We used water, Grace’s insect medium, and OptiMEM medium as negative controls. The ionophore ionomycin (Thermo Fisher Scientific) was used as a positive control (10 mM stock solution in water, 100 μM in the assay). The complexes were added to supplemented L-15 medium without penicillin/streptomycin to avoid antibiotic-related cytotoxicity and the plates were incubated at 28 °C. The medium was replenished after 6 h using supplemented medium with 1% penicillin/streptomycin. At 48 h post-treatment (hpt), cell viability was assessed by measuring ATP levels using the CellTiter-Glo Luminescent Cell Viability assay (Promega, Walldorf, Germany), according to the manufacturer’s instructions. Luminescence was recorded using black 96-well plates in a Cytation 5 Cell Imaging Multimode Reader (Agilent Technologies, Waldbronn, Germany). The data were normalized to the untreated control and expressed as percentage (treatment/untreated × 100).

**Table 2:** List of transfection reagents used and their determined optimal complexing ratio as well as the volume of reagent required to completely complex 400 ng mCherry-dsRNA. The ratio is expressed as dsRNA:TR (w/w)

Transfection reagent	Optimal ratio	Volume of TR required (µL)
Metafectene Pro	1:0.7	0.19
K4 transfection system	1:1	0.27
Metafectene SI +	1:1.5	0.40
Lipofectamine 2000	1:3	1.20
CellFectin II Reagent	1:5	2.00
Lipofectamine RNAiMAX	1:7	2.80
SiLentFect Lipid Reagent	1:7	2.80
HiPerFect Transfection Reagent	> 1:9	> 3.60

The * ratio is expressed as dsRNA:TR (w/w)

### Stability of dsRNA in cell culture supernatant

Supernatant was collected from C6/36 and U4.4 cells at 80–100% confluency. The mCherry-dsRNA was diluted to 0.4 µg/µL and 1 µL of dsRNA was incubated for 20 or 240 min at 28 °C with 10 µL of supernatant. Water, unsupplemented L-15 medium, and fresh supplemented L-15 medium were used as negative controls. RNase III (New England BioLabs) was used as a positive control for dsRNA cleavage. Immediately after incubation, the samples were mixed with Mass Ruler loading dye and resolved by 2% agarose gel electrophoresis for 35 min at 110 V and 150 mA. The gel was visualized as described above.

### Uptake of dsRNA into C6/36 and U4.4 cells

The mCherry-dsRNA was labeled with Cy3 using the Silencer siRNA labeling kit (Thermo Fisher Scientific) according to the manufacturer’s protocol. C6/36 and U4.4 cells were seeded in black F-bottom µclear 96-well plates (Greiner Bio-One, Frickenhausen, Germany) and transfected at a confluency of 80% with 50 or 200 ng of labeled dsRNA using K4, Metafectene Pro, Metafectene SI+, Lipofectamine 2000, or CellFectin II at the determined optimal complexation ratio (Table 2). Uncomplexed labeled dsRNA was added to the wells as control. At 6 hpt, cells were washed three times with unsupplemented L-15 medium and replenished with fresh supplemented medium. At 24 hpt, we added 8 µL of Hoechst 33,342 (NucBlue Live ReadyProbes Reagent, Thermo Fisher Scientific) per well and incubated the cells for 30 min at 28 °C. We monitored the Cy3 and Hoechst 33342 fluorescence signals using a Cytation 5 Cell Imaging Multimode reader. We captured bright-field images of each well at 20× magnification as well as fluorescence images using DAPI and Texas red filters. The images were processed using BioTek Gen5 Image Prime v3.12 (Agilent Technologies). The raw fluorescence signal of the untransfected control was subtracted from the treatments.

### Virus production

BHK-21 cells at 90% confluency were transfected with the SFV6-2SG-mCherry plasmid (kindly provided by Prof. Dr. Andres Merits and Prof. Dr. Andreas Pichlmair) in infection medium [DMEM Glutamax supplemented with 1% penicillin/streptomycin and 0.2% bovine serum albumin (BSA; Serva Electrophoresis, Heidelberg, Germany)] using Lipofectamine 3000 (Thermo Fisher Scientific) according to the manufacturer’s instructions. At 48 hpt, the virus-containing supernatant was collected, frozen in aliquots and stored at −80 °C prior to the infection of BHK-21 cells in T-75 flasks (Greiner Bio-One) to produce the virus stocks. Titers were determined in BHK-21 cells using a TCID_50_ assay. Briefly, tenfold serial dilutions of each sample were inoculated with the cells, which were incubated for 1 h as described above. After infection, cells were incubated for 48 h at 37 °C in a 5% CO_2_ atmosphere before virus replication was quantified by fluorescence analysis.

### Efficacy of transfection reagents in C6/36 and U4.4 cells

Cells were seeded in a black F-bottom µclear 96-well plates and cultivated as described above until they reached ~50% confluency. The cells were transfected with dsRNAs (0.5 ng/µL) targeting GFP or mCherry using the TRs with the optimal complexing ratio (Table 2) in supplemented L-15 medium without penicillin/streptomycin. At 6 hpt, the medium was replaced with fresh supplemented L-15 medium including 1% penicillin/streptomycin. Cells at a confluency of 80% were infected 24 hpt with mCherry-SFV at a multiplicity of infection (MOI) of 0.01 using unsupplemented L-15 medium. The medium was replaced with supplemented L-15 medium after 1 h. At 30 h (for C6/36) and 56 h (for U4.4) post-infection (hpi), we added 8 µL of NucBlue Live ReadyProbes Reagent per well and incubated the cells for 30 min at 28 °C. We captured images of each well at 4× magnification using bright-field, DAPI, and Texas red filters as described above. The images were processed using BioTek Gen5 Image Prime v3.12 to determine the total intensity of red fluorescence per cell (total red intensity/cell count).

### Statistical analysis

The statistical analysis and visualization of data was carried out using GraphPad Prism v9.5.1 (GraphPad Software, Boston, MA, USA). We used one-way analysis of variance (ANOVA) with Dunnett’s or Šidák’s multiple comparisons tests to determine the statistical significance of any differences in the efficacy between TRs (*P* < 0.05).

## Results

### Complexing capacity of the selected transfection reagents

To develop a protocol for the efficient transfection of aedine cells with long dsRNA, we compared the TRs K4, Metafectene Pro, Metafectene SI+, Lipofectamine 2000, Lipofectamine RNAiMAX, CellFectin II, SiLentFect, and HiPerFect. The complexing capacity of each TR was analyzed by agarose gel electrophoresis using the concentrations recommended by the manufacturers and 400 ng of dsRNA with a length of 409 bp to determine the optimal dsRNA:TR ratio. The TRs varied in their complex-formation capacity over a wide range. Metafectene Pro, K4, and Metafectene SI+ formed complexes most efficiently, with dsRNA:TR ratios of 1:0.7, 1:1, and 1:1.5, respectively (Table 2 and Additional file 1: Fig. S1). Lipofectamine 2000 complexed the same amount of dsRNA at a ratio of 1:3, whereas CellFectin II required a ratio of 1:5 and both Lipofectamine RNAiMAX and SiLentFect required a ratio of 1:7. Uniquely, HiPerFect was unable to complete the formation of complexes even at a ratio of 1:9. Based on these results, we excluded Lipofectamine RNAiMAX, SiLentFect, and HiPerFect from further experiments.

### Toxicity of dsRNA/TR complexes in aedine cells

**Fig. 1:**
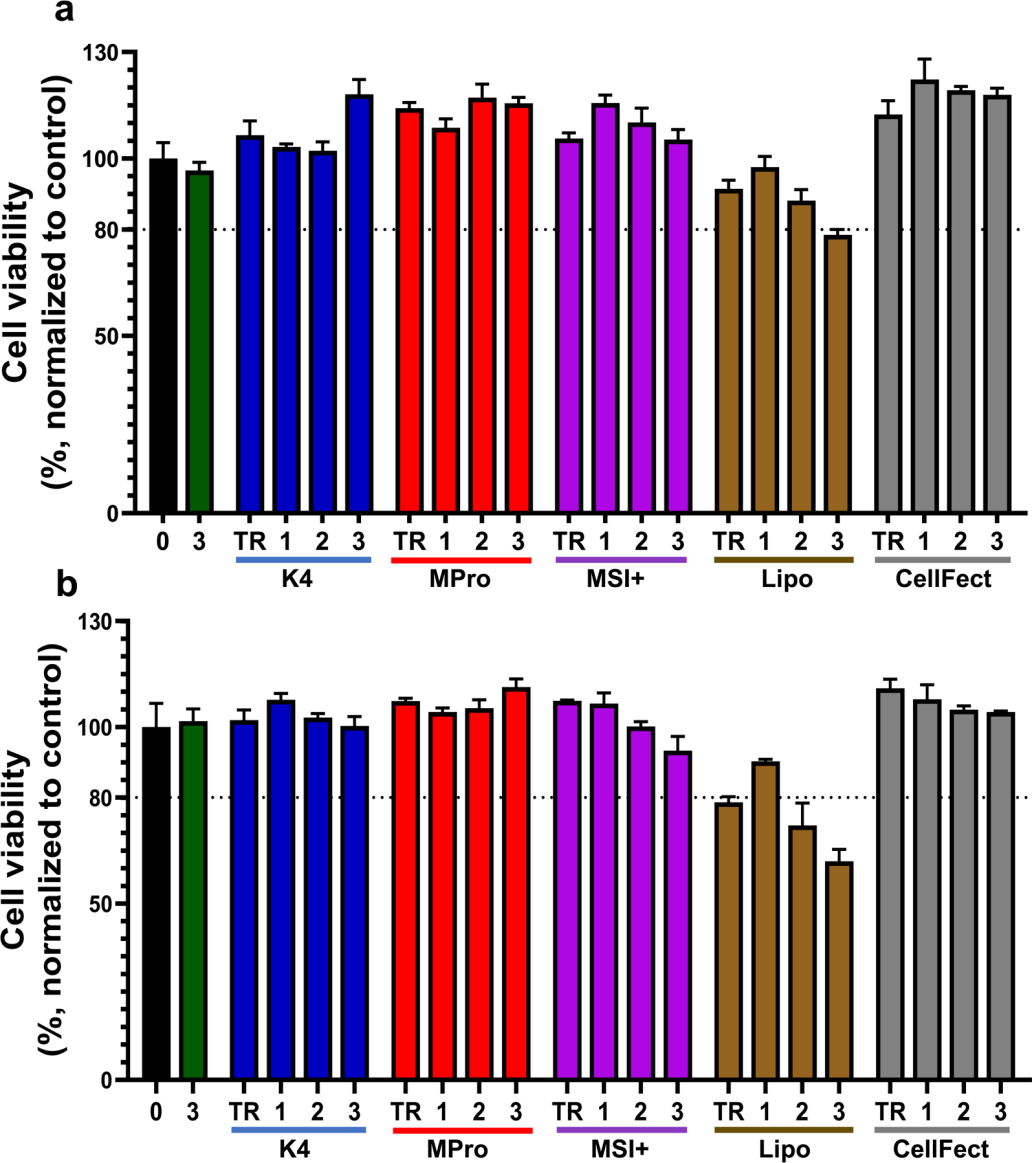
Cytotoxicity of dsRNA complexed with five transfection reagents in *Ae. albopictus* (**a**) C6/36 and (**b**) U4.4 cells. The cells were treated at ~50% confluency with only the TR or with (1) 50, (2) 100, and (3) 200 ng of the complexes of TRs and mCherry-dsRNA per well (*n* = 6). Cell viability was determined using a CellTiter-Glo assay. The data were normalized to the untreated control (treatment/control × 100) and the mean cell viability is displayed, with error bars representing coefficient of variation (both in %). The untreated control is represented as 0. The dotted line represents the toxicity cutoff set at 80%. Abbreviations: *K4* K4 transfection system, *MPro* Metafectene Pro, *MSI+* Metafectene SI+, *Lipo* Lipofectamine 2000, *CellFect* CellFectin II

The toxicity of the five remaining TRs and their lipoplexes at the optimal dsRNA:TR ratios were determined in aedine cells by measuring the abundance of ATP using the CellTiter-Glo assay. The TRs were used to introduce 50–200 ng mCherry-dsRNA into C6/36 and U4.4 cells. None of the lipoplexes showed any significant toxicity against C6/36 cells (all values exceeded the toxicity threshold of 80% viability). Only the transfection with 200 ng mCherry-dsRNA using Lipofectamine 2000 was toxic, reducing cell viability to 78.5% (Fig. 1a). The same trend was observed in U4.4 cells. Here, Lipofectamine 2000 alone and in complexes with 100 or 200 ng of mCherry-dsRNA were toxic, reducing cell viability to 78.7%, 72.1%, and 62%, respectively (Fig. 1b).

### Effect of the transfection reagents on the uptake of dsRNA into aedine cell lines

To ensure that the mCherry-dsRNA is not degraded before it is taken up by the cells, we tested its stability in the culture supernatant of C6/36 and U4.4 cells. We incubated the dsRNA in the supernatant for 20 and 240 min before analysis by agarose gel electrophoresis. RNase III was added to the mCherry-dsRNA as positive control for degradation. We observed no substantial degradation of the dsRNA in the cell culture supernatant at either of the time points (Fig. S2).

**Fig. 2:**
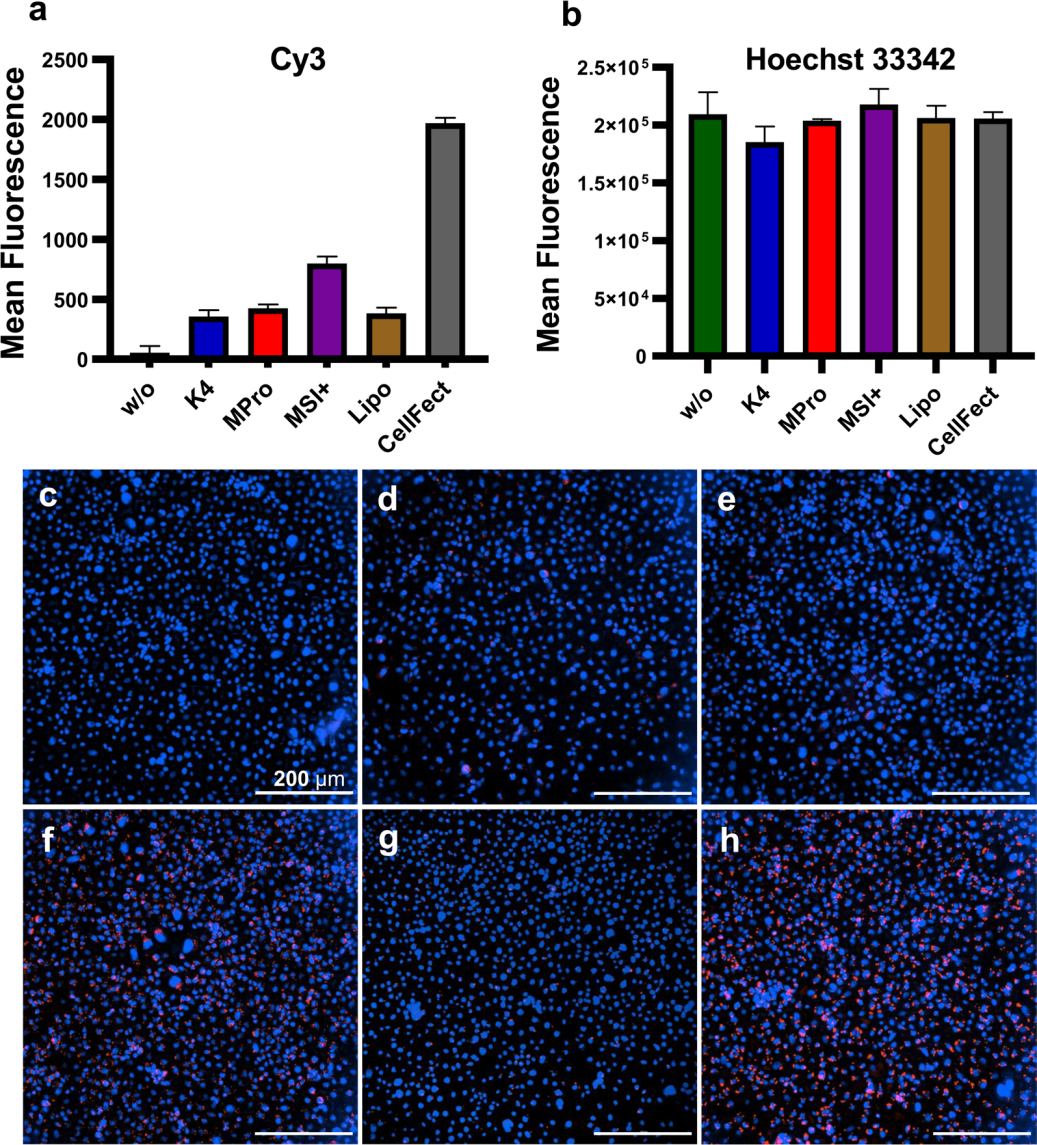
Uptake of Cy3-labeled dsRNA into C6/36 cells 24 h post-transfection. **a** Mean fluorescence of Cy3 signal from the labeled dsRNA and (**b**) mean fluorescence of Hoechst 33342 (both *n* = 3, with error bars indicating standard deviations). **c**–**h** Fluorescence images taken at 20× magnification. **c** Labeled dsRNA was applied without transfection reagent (w/o). **d**–**h** Cells were transfected with the labeled dsRNA using (**d**) K4, (**e**) Metafectene Pro, (**f**) Metafectene SI+, (**g**) Lipofectamine 2000, and (**h**) CellFectin II. The TRs were used to transfect cells with 200 ng labeled dsRNA, except Lipofectamine 2000 with only 50 ng. The cell nuclei are stained in blue and the labeled dsRNA in red. The percentage of transfected cells per image for all conditions can be found in Table S2

**Fig. 3:**
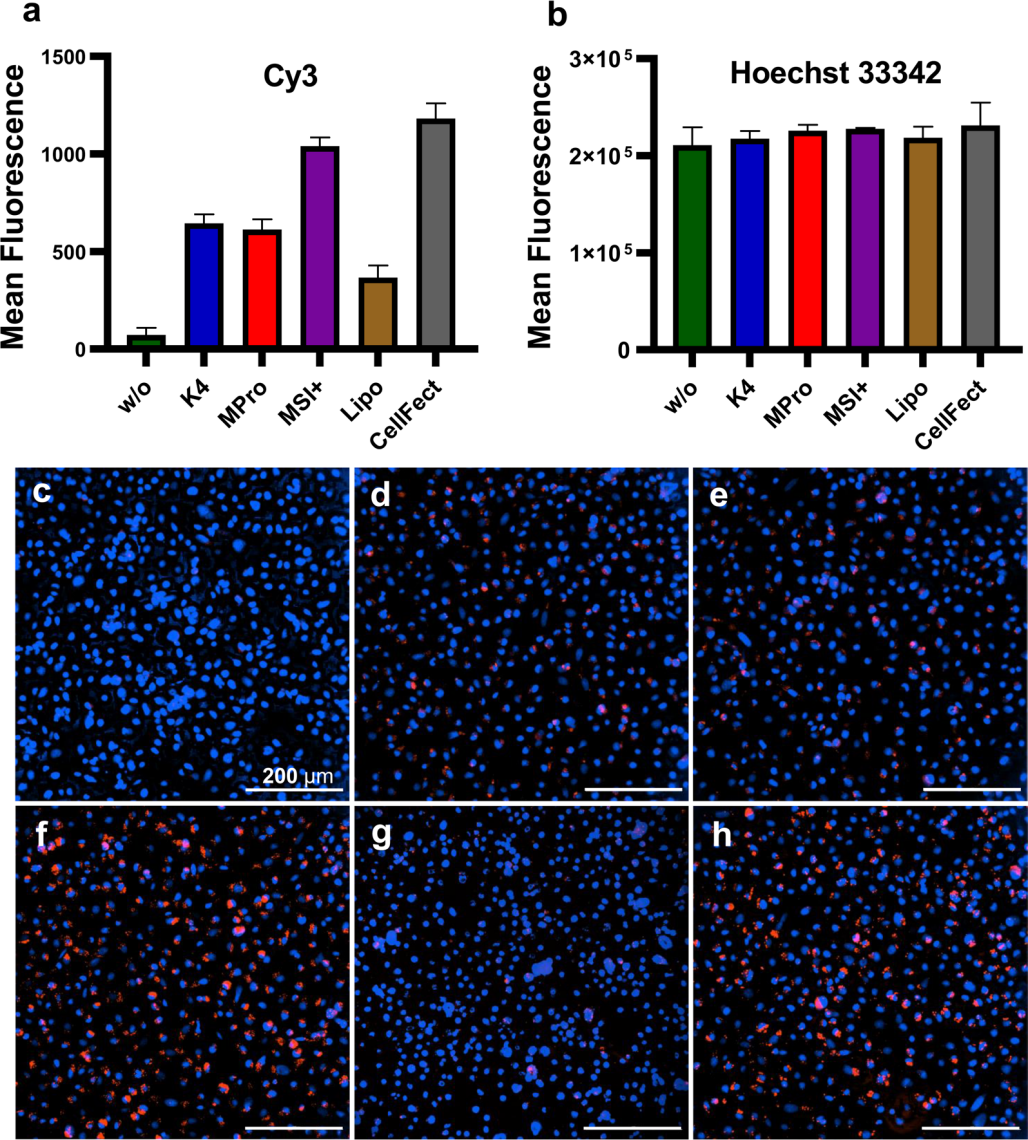
Uptake of Cy3-labeled dsRNA into U4.4 cells 24 h post-transfection. **a** Mean fluorescence of Cy3 signal from the labeled dsRNA and (**b**) mean fluorescence of Hoechst 33342 (both *n* = 3, with error bars indicating standard deviations). **c**–**h** Fluorescence images taken at 20× magnification. **c** Labeled dsRNA was applied without transfection reagent (w/o). **d**–**h** Cells were transfected with the labeled dsRNA using (**d**) K4, (**e**) Metafectene Pro, (**f**) Metafectene SI+, (**g**) Lipofectamine 2000, and (**h**) CellFectin II. The TRs were used to transfect cells with 200 ng labeled dsRNA, except Lipofectamine 2000 with only 50 ng. The cell nuclei are stained in blue and the labeled dsRNA in red. The percentage of transfected cells per image for all conditions can be found in Table S2

To study the uptake of dsRNA by aedine cells, mCherry-dsRNA was labeled with the fluorophore Cy3 and introduced into the cells using each of the five TRs. In most cases we transfected the cells with 200 ng of labeled dsRNA, but only 50 ng was used with Lipofectamine 2000 because cytotoxicity was observed at higher concentrations (Fig. 1). The fluorescence intensity following transfection varied among the five reagents, with CellFectin producing the strongest signal, followed by Metafectene SI+ in both C6/36 cells (Fig. 2) and U4.4 cells (Fig. 3). The signal from the cells treated with naked dsRNA was negligible. The Hoechst 33342 signal from the stained nuclei was comparable among the different treatments (Figs. 2b, 3b).

### Knockdown of the virus reporter mCherry in aedine cell lines using long dsRNA

**Fig. 4:**
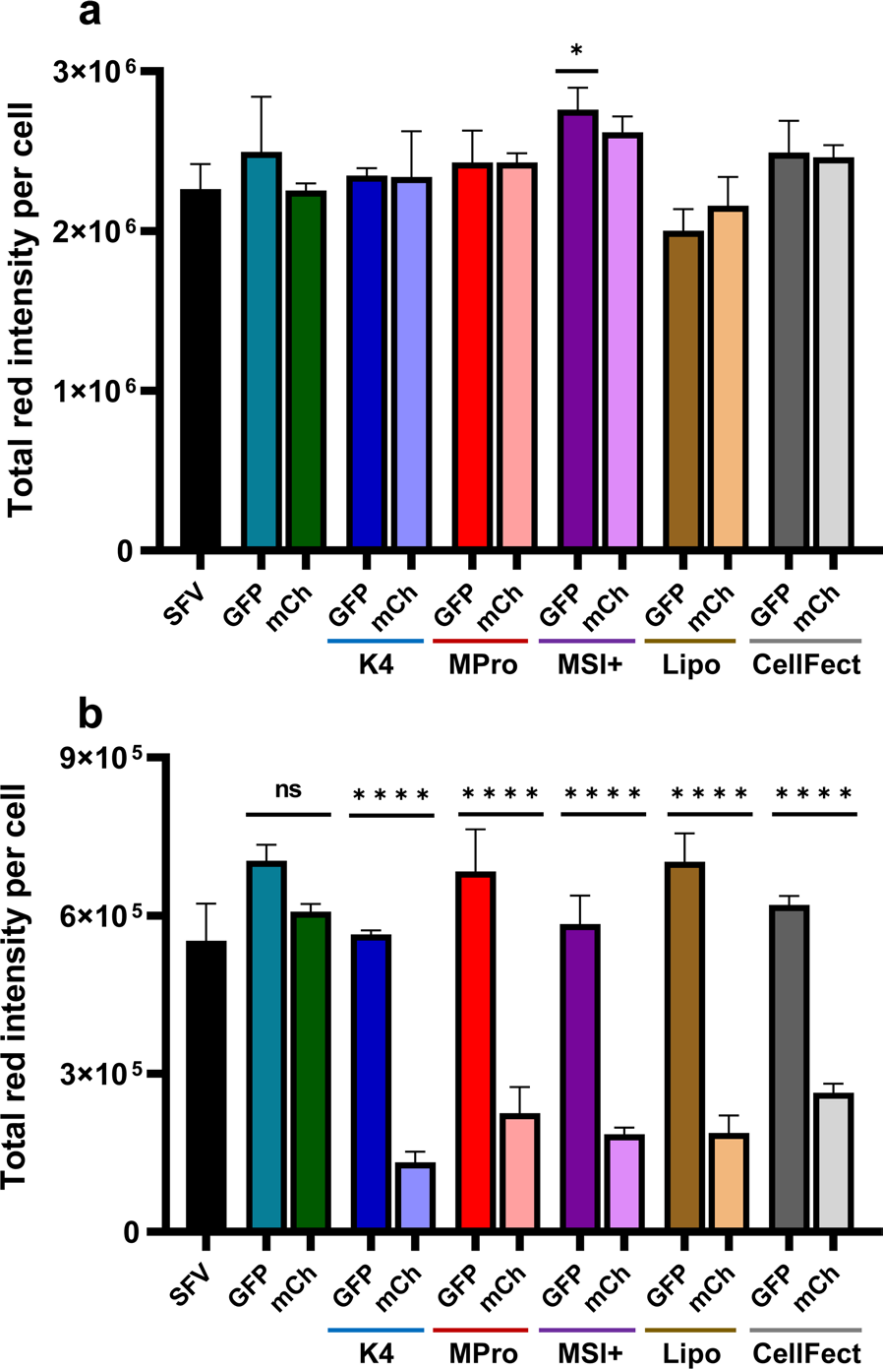
Knockdown of virus reporter mCherry-SFV in (**a**) C6/36 and (**b**) U4.4 cells. Both cell lines were treated with 0.5 ng/µL dsRNA targeting GFP or mCherry (mCh). The dsRNAs were introduced into the cells using K4, Metafectene Pro (MPro), Metafectene SI+ (MSI), Lipofectamine 2000 (Lipo), or CellFectin II (CellFect). At 24 hpt, cells were infected with mCherry-SFV. The total intensity and cell count were analyzed at 30 (for C6/36) and 56 (for U4.4) hpi. The data are mean values (*n* = 3) of the total red intensity per cell (total red intensity/total cell count) and the error bars represent standard deviations. Statistical significance was determined by ANOVA and Šidák’s multiple comparison test (****P < 0.0001, ns = *P* > 0.05)

To study the knockdown of the reporter virus SFV-mCherry and the RNAi response of C6/36 and U4.4 cells, we transfected both cell lines with 0.5 ng/µL of dsRNA targeting the reporter gene mCherry using K4, Metafectene Pro, Metafectene SI+, Lipofectamine 2000, and CellFectin II. To ensure that the observed knockdown effects resulted from an RNAi response, we used GFP-dsRNA as a control. We infected both cell lines with the mCherry-tagged SFV 24 hpt and acquired images showing the intensity of mCherry fluorescence and the total cell count at 30 (for C6/36) and 56 (for U4.4) hpi (Fig. 4). We observed no RNAi response in C6/36 cells, resulting in no significant differences between the treatments and control, except for the GFP-dsRNA transfection with Metafectene SI+ (*P* = 0.0156). In contrast, U4.4 cells showed a potent RNAi response, resulting in the significant knockdown of reporter mCherry expression in the transfected cells. Cells treated with naked dsRNA showed no significant knockdown effects. The most efficient TR was K4, followed by Metafectene SI+ , Lipofectamine 2000, Metafectene Pro, and CellFectin II. However, Šidák’s multiple comparisons test revealed no significant differences in the ability of the TRs to knock down mCherry expression in U4.4 cells.

## Discussion

We compared eight TRs to determine their complexing capacity, cytotoxicity, impact on the uptake of dsRNA, and efficacy in two *Ae. albopictus* cell lines (C6/36 and U4.4), revealing the optimal dsRNA:TR ratios and concentrations that are compatible with these aedine cells*.* In addition to establishing these optimal transfection conditions, we also found evidence supporting previous claims that C6/36 cells lack a functional RNAi pathway.

Cell culture experiments can provide initial evidence to guide the application of RNAi in vivo [27, 44]. The use of TRs improves the cellular uptake of dsRNA by protecting it from enzymatic degradation as well as pH changes, which is necessary to ensure that an adequate amount of dsRNA reaches the cytoplasm to trigger RNAi [42, 46]. However, the composition and formulation of many TRs is unclear because the information is not publicly available [40]. Additionally, most protocols focus on the transfection of cells with siRNA and plasmids [47, 48]. To our knowledge, there have been no comparative studies on the transfection of aedine cell lines with long dsRNA using TRs. Furthermore, there are no commercially available TRs specifically designed for long dsRNA. Therefore, it is necessary to establish protocols allowing the evaluation and optimization of commercially available TRs for the introduction of long dsRNA into aedine cell lines.

Accordingly, we used agarose gel electrophoresis to determine the complexing capacity of TRs and the optimal dsRNA:TR ratio (the lower the ratio, the better the complexing capacity and vice versa). The TRs from Biontex (K4, Metafectene Pro, and Metafectene SI+) showed the best complexing capacity with the lowest complexing ratios (Table 2 and Additional file 1: Fig. S1). Low ratios are advantageous because they reduce the potential for cytotoxicity by limiting the amount of TR needed for efficient transfection, also reducing costs, especially when large numbers of transfection experiments are required. Impressively, the complexing ratio of TRs from Biontex was lower than the ranges recommended by the manufacturer in their protocol (1:2–1:7) for transfection with DNA and RNA. In contrast, HiPerFect was the only TR that was unable to form complete complexes with the same amount of dsRNA even at a ratio of 1:9. This may reflect the specific design of HiPerFect for the transfection of cells with siRNA, miRNA mimics, and inhibitors (according to the manufacturer’s information), potentially making it unsuitable for long dsRNA. Furthermore, for the Biontex TRs we relied on TR concentrations recommended by the manufacturer but not included in the instructions (Biontex, personal communication). For Lipofectamine RNAiMAX and HiPerFect, the manufacturers did not provide the concentrations of the TRs. The method we used to study the complexing capacity of the TRs has already been used to determine the optimum ratio of carrier systems, such as cationic polymers, nanoparticles, and TRs, for the complexation of nucleic acid molecules [46, 49, 50]. However, to the best of our knowledge, this method has only been used in one study to evaluate the encapsulation of long dsRNA, revealing that 1 µL of GenJet is required to completely complex 0.5 µg of the long dsRNA-targeting eGFP [46].

The commercially available TRs were specifically designed to deliver genetic material into cells but may have inherent cytotoxic effects [51, 52]. The viability of transfected cells is an important parameter for the accurate interpretation of RNAi results because it is necessary to distinguish between potential effects caused by RNAi and general cytotoxicity [51]. Most of the TRs we tested did not significantly affect cell viability at the concentrations we used, with the exception of Lipofectamine 2000 at high concentrations (Fig. 1). This is one of the most commonly used TRs in mammalian cells and consists of 1,2-dioleoyl-*sn*-glycero-3-phosphatidylethanolamine (DOPE) and 2′-(1′′,2′′-dioleoyloxypropyldimethyl-ammonium bromide)-*N*-ethyl-6-amidospermine tetratrifluoroacetic acid salt (DOSPA) formulated with a helper/neutral co-lipid [53]. Lipofectamine 2000 has previously been shown to affect the viability of Huh-7 liver cancer cells, SHSY5Y neuroblastoma cells, JU77 lung mesothelioma cells, HL60 promyelocytic leukemia cells, HEK293 embryonic kidney cells, and U87MG brain cancer cells, when used to introduce single-stranded oligonucleotides (SSOs) at an SSO:TR ratio of 1:2 [51]. Similarly, a 31.9% reduction in cell viability was reported when Lipofectamine 2000 was used to transfect mouse protoblast MC3T3-E1 cells with plasmids encoding luciferase or β-galactosidase [41].

The uptake of dsRNA into cells is facilitated by TRs, thus enhancing RNAi efficacy [54]. For this reason, we also compared the TRs for their effects on the uptake of dsRNA into C6/36 and U4.4 cells. We found that CellFectin II facilitated the uptake of Cy3-labeled dsRNA most efficiently in both C6/36 and U4.4 cells after 24 h, followed by Metafectene SI+ (Figs. 2, 3). Only minimal amounts of the Cy3-labeled dsRNA were taken up in the absence of a TR, confirming the ability of TRs to significantly improve the uptake of nucleic acids [54, 55]. CellFectin II was the only TR we tested that is specifically designed for the transfection of insect cells, according to the manufacturer’s information. It has been used for the transfection of *Drosophila* S2 cells with DNA [56] and for the in vivo transfection of adult-stage *Ae. aegypti* with plasmid DNA targeting *Ae. aegypti* thioester-containing protein-1 (AeTEP-1), significantly inhibiting the infectivity of DENV [48].

Any TR that does not release a substantial amount of dsRNA in the cytoplasm is unlikely to initiate an effective RNAi response [57]. We therefore analyzed the TRs based on their ability to knock down the virus reporter mCherry by transfecting aedine cells with mCherry-dsRNA and then infecting them with the mCherry-SFV. We observed a substantial knockdown of the virus reporter in U4.4 cells transfected with mCherry-dsRNA using K4, Metafectene Pro, Metafectene SI+, Lipofectamine 2000, and CellFectin II (Fig. 4). We found no substantial differences among the TRs in terms of knockdown efficacy. In a previous study, Lipofectamine 2000 was compared with ExGen 500, TurboFectin 8.0, and PrimeFect I DNA, which were used to transfect mammalian cell lines NG108-15, SH-SY5Y, and CHO-K1 with DNA encoding GFP at a DNA:TR ratio of 1:2.5. Lipofectamine 2000 was 11-fold more efficient than TurboFectin 8.0 and was preferred in terms of transfection efficiency for NG108-15 and CHO-K1 cells [58]. On the other hand, in an experiment using *Ae. aegypti* adult females, CellFectin II was used to introduce long dsRNA targeting p400. CellFectin II only improved the knockdown efficiency when complexed with 2 µg of dsRNA, which was then injected into the mosquito. However, there was no significant knockdown of p400 when the complex contained either only 0.5 or 1 µg of the same dsRNA [59].

Most RNAi research on the control of mosquitos has focused on *Ae. aegypti* while neglecting *Ae. albopictus*, a competent vector of more than 22 arboviruses including ZIKV, DENV, and CHIKV [25, 60]. Candidate dsRNAs are usually screened in cell-based assays [61], but C6/36 cells, the most commonly used *Ae. albopictus* cell line, appear to have a dysfunctional RNAi pathway [45]. On the other hand, U4.4 cells are widely used for host–virus interaction studies [62] and show a normal RNAi response [28]. Because of this suspected dissimilarity, we compared C6/36 and U4.4 cell lines to determine their antiviral RNAi responses. We observed no significant knockdown of the virus reporter mCherry in transfected C6/36 cells (Fig. 4), whereas there was a significant difference between transfected and untransfected U4.4 cells in terms of viral replication. Furthermore, viral replication was faster and generated much higher titers in C6/36 compared to U4.4 cells (Fig. S3). These findings support the reported dysfunction of the antiviral RNAi response in C6/36 cells, preventing the suppression of viral replication [27, 45]. The infection of C6/36 cells and S2 cells (control cells from *Drosophila melanogaster*) with WNV, Sindbis virus (SINV), and La Crosse virus (LACV) resulted in the production of viral interfering RNA (viRNA) only 17 nt in length from WNV compared to 26–27 nt from SINV and LACV in C6/36 cells. In contrast, all three viruses induced the production of 21-nt viRNAs in S2 cells [45]. This suggests that C6/36 cells lack the capacity to process long dsRNAs into siRNAs that can be used by the RNAi machinery [27].

The testing of TRs in cell lines helps to establish conditions that are suitable for in vivo applications involving the introduction of siRNA, dsRNA, and plasmids [48]. The first TR used for the in vivo transfection of mosquitos was Effectene, allowing the delivery of dsRNA targeting MAPK p38 in *Ae. aegypti* larvae [34]. Cellfectin II was subsequently used to deliver plasmid DNA intrathoracically into adult stage *Ae. aegypti* and *Anopheles gambiae* [48]. Some toxic effects were also reported: for example, FuGene 6 was highly toxic to *Ae. aegypti*, with only one of 120 injected mosquitos surviving, whereas Cellfectin II was well tolerated under the same conditions, with 99% survival [48]. Similarly, RNA-free Effectene liposomes caused 5% mortality in *Ae. aegypti* larvae [34].

We analyzed the cytotoxicity, dsRNA uptake and overall efficacy of TRs according to their complexing capacity (Table 2), aiming to minimize the excess of TRs needed for the transfection of aedine cells. Consequently, our results may not be directly comparable because different dsRNA:TR ratios were used for each reagent. In summary, for the transfection of aedine cell lines, Lipofectamine 2000 improved dsRNA uptake and enhanced the knockdown against our target, but a low concentration is required to avoid cytotoxic effects. CellFectin II achieved the highest dsRNA uptake with no cytotoxicity and also led to the significant knockdown of our target, but the high dsRNA:TR ratio makes it less cost effective. K4, Metafectene Pro, and Metafectene SI+ are good candidates for further studies due to their high complexing capacity, absence of cytotoxicity, ability to promote the uptake of dsRNA, and efficient knockdown of the virus reporter mCherry.

## Conclusions

In this study, we comprehensively compared eight TRs based on their complexing capacity, cytotoxicity, impact on the uptake of dsRNA, and efficacy in two *Ae. albopictus* cell lines (C6/36 and U4.4). Our data support previous studies reporting that C6/36 cells have a dysfunctional antiviral RNAi response, given the substantial differences we observed between the C6/36 and U4.4 cells. Our findings will facilitate RNAi research for the analysis of gene functions as well as vector control and will serve as a basis for the rational selection of TRs for future experiments in aedine cell lines.

### Supplementary Information

**Additional file 1.**

## Data Availability

All data generated or analyzed during this study are included in this published article.
