# Supplementary material for: Gene silencing in the aedine cell lines C6/36 and U4.4 using long double-stranded RNA
Source: Parasit Vectors. 2024 Jun 11;17:255. doi: 10.1186/s13071-024-06340-3 (PMC11167938; doi:10.1186/s13071-024-06340-3)
Supplement: Supplementary file 1 — Additional file 1. [file 13071_2024_6340_MOESM1_ESM.docx]

**Additional file 1**

**Gene silencing in the aedine cell lines C6/36 and U4.4 using long double-stranded RNA**

**Bodunrin Omokungbe^1,2^, Alejandra Centurión ^1,3^, Sabrina Stiehler^2^, Antonia Morr^3^, Andreas Vilcinskas^1,2,3^, Antje Steinbrink^1,2^, Kornelia Hardes^1,3,4^**

^1^ LOEWE Centre for Translational Biodiversity Genomics (LOEWE TBG), Senckenberganlage 25, 60325 Frankfurt am Main, Germany

^2^ Institute for Insect Biotechnology, Justus-Liebig University, Heinrich-Buff-Ring 26-32, 35392 Giessen, Germany

^3^ Fraunhofer Institute for Molecular Biology and Applied Ecology IME, Branch of Bioresources, Ohlebergsweg 12, 35392 Giessen, Germany

^4^ BMBF Junior Research Group in Infection Research „ASCRIBE” Ohlebergsweg 12, 35392 Giessen, Germany

Table S1: List of primers for the synthesis of dsRNA and dsRNA template sequences.

| Primer sequence | |
| --- | --- |
| Name | Sequence 5′→3′ |
| mCherrydsR-T7-FW | TAATACGACTCACTATAGGGGCGTGATGAACTTCGAGGAC |
| mCherrydsR-T7-RV | TAATACGACTCACTATAGGGCTTGTACAGCTCGTCCATGC |
| GFP-T7-FW | CCCTTTAATACGACTCACTATAGGGAGAACCACATGAAGCAGCACGACTT |
| GFP-T7-RV | CCCTTTAATACGACTCACTATAGGGAGAGTCCATGCCGAGAGTGATCCCG |
| Template sequence | |
| mCherry-dsRNA | CGTGATGAACTTCGAGGACGGCGGCGTGGTGACCGTGACCCAGGACTCCTCCCTGCAGGACGGCGAGTTCATCTACAAGGTGAAGCTGCGCGGCACCAACTTCCCCTCCGACGGCCCCGTAATGCAGAAGAAGACCATGGGCTGGGAGGCCTCCTCCGAGCGGATGTACCCCGAGGACGGCGCCCTGAAGGGCGAGATCAAGCAGAGGCTGAAGCTGAAGGACGGCGGCCACTACGACGCTGAGGTCAAGACCACCTACAAGGCCAAGAAGCCCGTGCAGCTGCCCGGCGCCTACAACGTCAACATCAAGTTGGACATCACCTCCCACAACGAGGACTACACCATCGTGGAACAGTACGAACGCGCCGAGGGCCGCCACTCCACCGGCGGCATGGACGAGCTGTACAAG |
| GFP-dsRNA (+T7) | CCCTTTAATACGACTCACTATAGGGAGAACCACATGAAGCAGCACGACTTCTTCAAGTCCGCCATGCCCGAAGGCTACGTCCAGGAGCGCACCATCTTCTTCAAGGACGACGGCAACTACAAGACCCGCGCCGAGGTGTAGTTCGAGGGCGACACCCTGGTGAACCGCATCGAGCTGAAGGGCATCGACTTCAAGGAGGACGGCAACATCCTGGGGCACAAGCTGGAGTACAACTACAACAGCCACAACGTCTATATCATGGCCGACAAGCAGAAGAACGGCATCAAGGTGAACTTCAAGATCCGCCACAACATCGAGGACGGCAGCGTGCAGCTCGCCGACCACTACCAGCAGAACACCCCCATCGGCGACGGCCCCGTGCTGCTGCCCGACAACCACTACCTGAGCACCCAGTCCGCCCTGAGCAAAGACCCCAACGAGAAGCGCGATCACATGGTCCTGCTGGAGTTCGTGACCGCCGCCGGGATCACTCTCGGCATGGACTCTCCCTATAGTGAGTCGTATTAAAGGG |


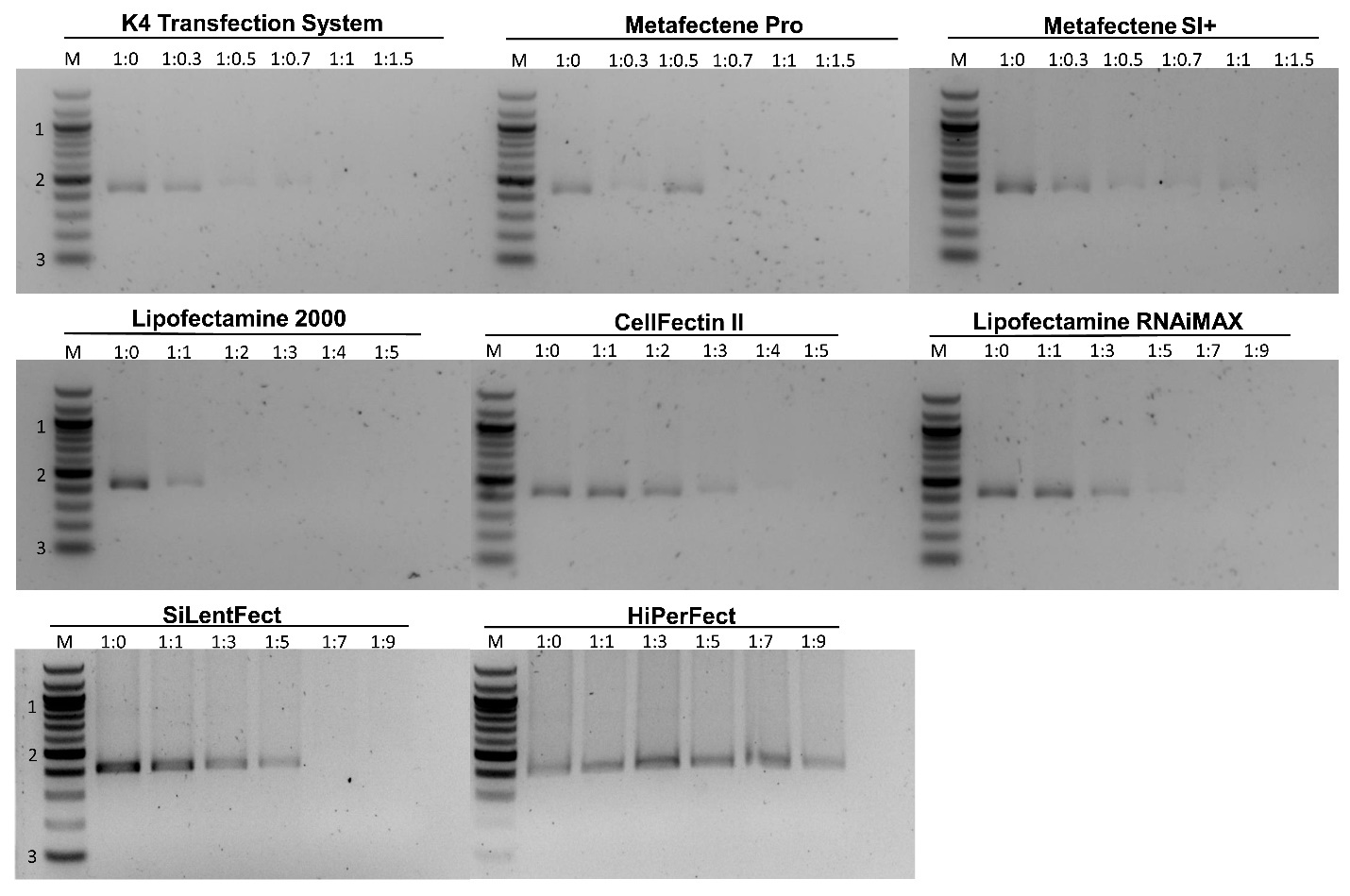


Fig. S1. Assessment of the complexing capacity of commercially available TRs using long dsRNA by incubating them at various ratios (dsRNA:TR) ranging from 1:0 to 1:9. Agarose gel electrophoresis was used to determine the point at which the TR completely complexed the dsRNA. M = 100 bp DNA Ladder (New England BioLabs); the numbers 1, 2 and 3 indicate the 1-kbp, 500-bp and 100-bp bands, respectively.


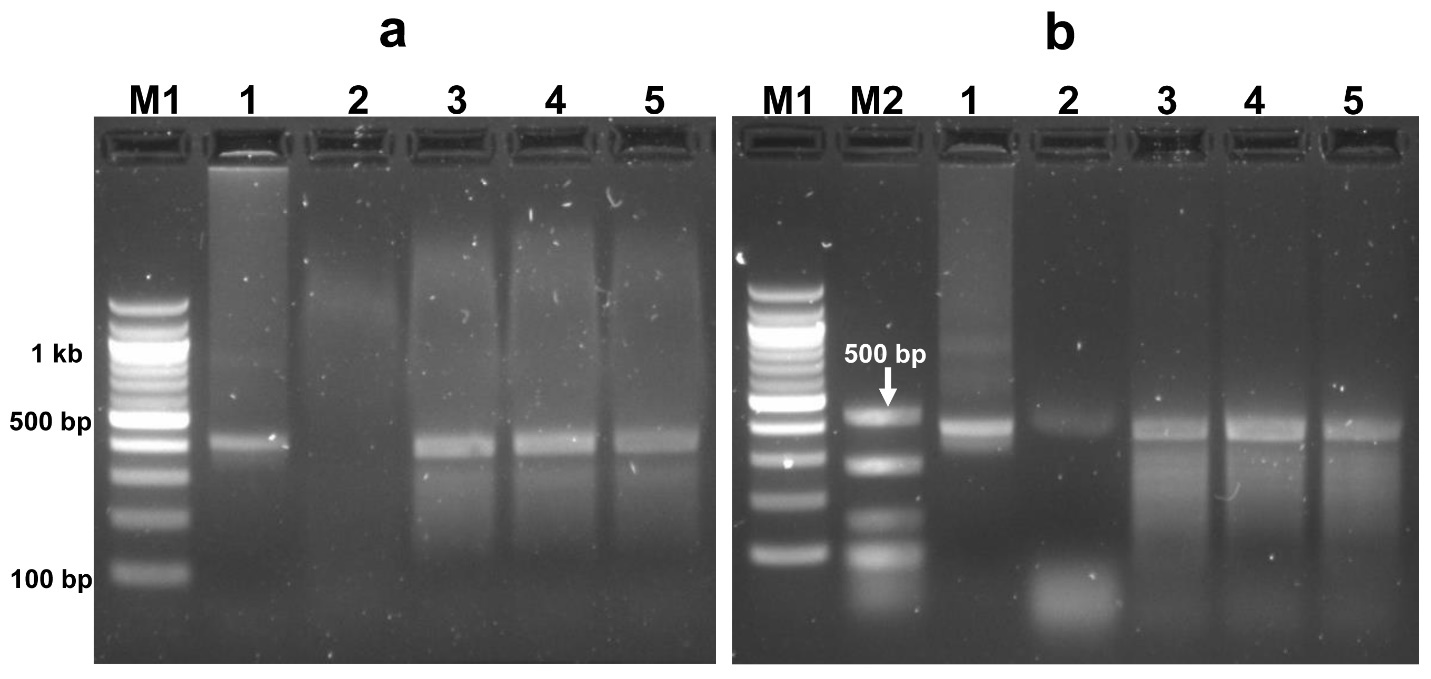


Fig. S2. Stability test of long dsRNA targeting mCherry in C6/36 and U4.4 cell supernatants. Agarose gel electrophoresis was used to determine the stability after incubation for 20 min (a) and 240 min (b). The dsRNA was incubated in 1: nuclease free water, 2: RNase III, 3: supplemented L-15 medium, 4: supernatant of C6/36 cell culture and 5: supernatant of U4.4 cell culture. The marker lanes are M1: 100 bp DNA Ladder and M2: dsRNA Ladder (both New England BioLabs).

Table S2. Uptake efficiency of dsRNA into C6/36 and U4.4 using TRs. Cells were transfected with labeled dsRNA using K4, Metafectene Pro, Metafectene SI+, Lipofectamine 2000, and CellFectin II. The TRs were used to transfect cells with 200 ng labeled dsRNA, except Lipofectamine 2000 with only 50 ng. Fluorescence pictures were taken at 24 hpt. The cells were counted using ImageJ v1.54d, the transfected cells were manually analyzed. The dsRNA uptake efficiency represents the ration of transfected cells to total cell count in percentage.

| Transfection reagent | dsRNA uptake efficiency (%) |
| --- | --- |
| C6/36 cell | |
| No Transfection reagent (w/o) | 3.09 |
| K4 transfection system | 95.52 |
| Metafectene Pro | 92.33 |
| Metafectene SI+ | 99.71 |
| Lipofectamine 2000 | 64.84 |
| CellFectin II | 97.24 |
| U4.4 cell | |
| No Transfection reagent (w/o) | 17.99 |
| K4 transfection system | 91.56 |
| Metafectene Pro | 90.22 |
| Metafectene SI+ | 98.48 |
| Lipofectamine 2000 | 59.83 |
| CellFectin II | 96.17 |


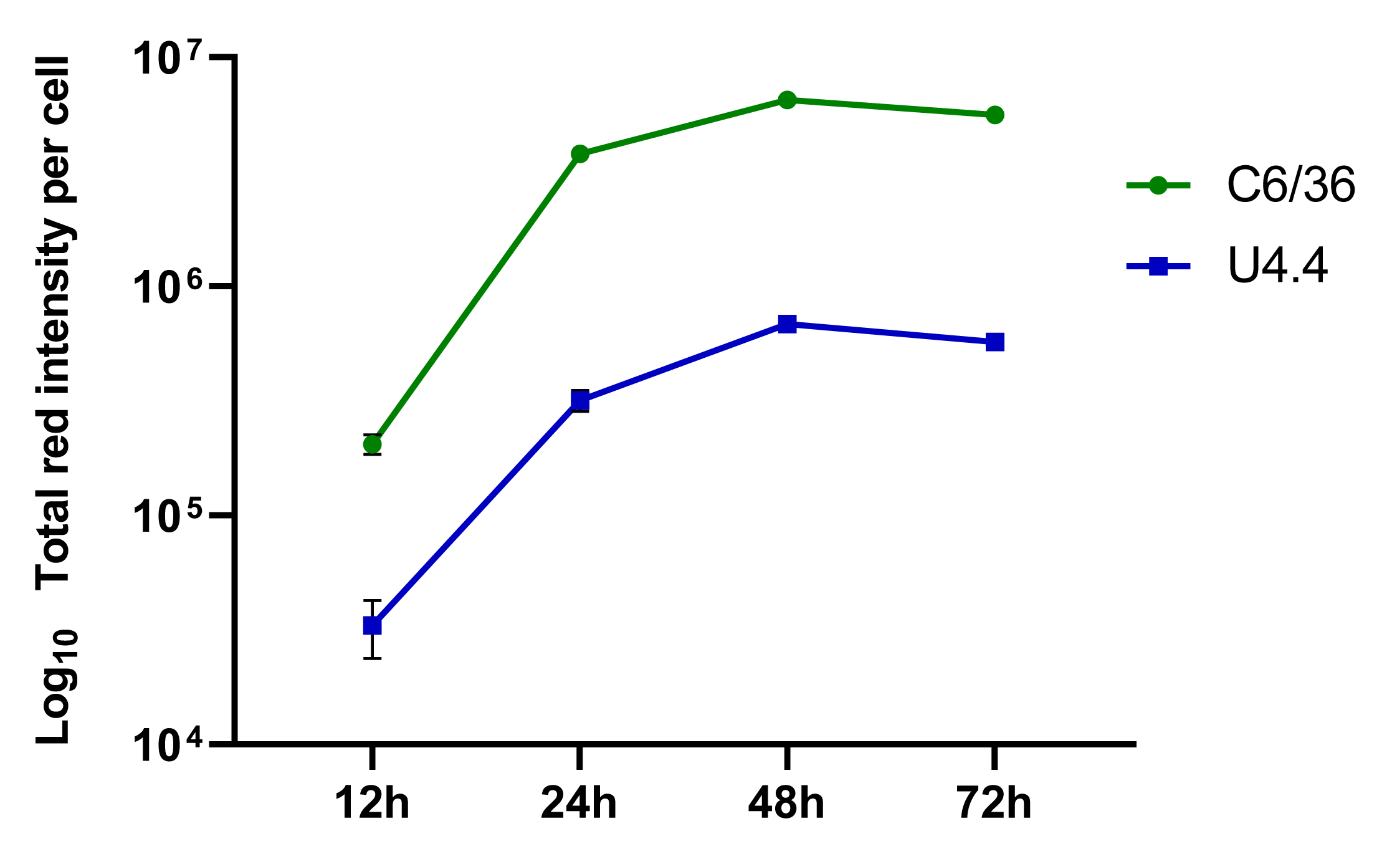


Fig. S3: Red fluorescence intensity in C6/36 and U4.4 cells after infection with mCherry-SFV. The cells were infected at full confluency with mCherry-SFV (MOI 0.01) and the total red intensity of the virus and cell count were analyzed at 12, 24, 48, and 72 hpi. The data are mean values (n = 3) of the total red intensity per cell (total red intensity/total cell count) and the error bars represent standard deviations.
